# Anaphylaxis after jellyfish ingestion with no history of stings: a pediatric case report

**DOI:** 10.1186/s13223-021-00601-x

**Published:** 2021-09-26

**Authors:** Mitsuru Tsuge, Masanori Ikeda, Osamu Mitani, Masato Yasui, Hirokazu Tsukahara

**Affiliations:** 1 Department of Pediatrics, Okayama University Graduate School of Medicine, Dentistry and Pharmaceutical Sciences, 2-5-1 Shikata-cho, Kita-ku, 700-8558 Okayama, Japan; 2 Department of Pediatrics, Fukuyama City Hospital, Fukuyama, Japan

**Keywords:** Food allergy, Anaphylaxis, Jellyfish, Immunoglobulin E, Basophil activation test, Oral food challenge, Skin prick test

## Abstract

**Background:**

Jellyfish stings are known to induce allergic skin reactions; however, case reports of anaphylaxis after jellyfish ingestion have been increasing, especially in Asian countries. Some cases of anaphylaxis after jellyfish ingestion have been reported in patients with a previous history of frequent jellyfish stings. Herein, we report a pediatric patient with anaphylaxis after jellyfish ingestion with no history of jellyfish stings.

**Case presentation:**

A 14-year-old girl developed two episodes of anaphylaxis, and her diet diaries revealed that edible jellyfish was common to the meals in both the anaphylaxis events. A skin prick test using five types of edible jellyfish products revealed a positive reaction to some jellyfish, and anaphylaxis was observed after the ingestion of jellyfish in an oral food challenge test. She had no history of jellyfish stings or frequent swimming in the ocean. The basophil activation test showed positive results on stimulation with extracts from various types of edible jellyfish. We observed serum immunoglobulin E (IgE) reactivity to purified jellyfish collagen and jellyfish acid-soluble extracts. Moreover, immunoblotting analysis showed IgE reactivity to two bands at approximately 40 and 70 kDa using purified jellyfish collagen, which may be a causative antigen.

**Conclusions:**

Edible salted jellyfish can be one of the causative foods of anaphylaxis. Clinicians should be aware of the possibility of anaphylactic reactions due to jellyfish ingestion even without a history of jellyfish stings.

## Background

Jellyfish are marine invertebrates belonging to the Scyphozoan class of the phylum Cnidaria, and it is estimated that there are 200 species of these animals. It is well known that jellyfish cause most marine envenomation worldwide. Jellyfish stings are known to induce immediate or delayed allergic skin reactions, and there are reports of allergic reactions caused by jellyfish stings [1–3].

Recently, case reports of anaphylaxis due to oral ingestion of edible jellyfish have been increasing in Asian countries [4]. Allergic reactions after salted jellyfish ingestion are rare, and there are seven case reports, including ours [5–10]. Jellyfish consumption has been part of the traditional Chinese food culture for centuries, and this practice has spread throughout Asia, such as Japan, Korea, and Southeast Asia. Recently, jellyfish has been attracting attention as a sustainable alternative food, in terms of cost, nutritional value, and availability, in response to future food shortages due to global population growth [11–13]. The consumption of jellyfish has already begun to spread to Western countries; therefore, with the worldwide spread of the dietary culture of jellyfish consumption, there is concern that the number of reports of anaphylaxis due to jellyfish ingestion will increase in the future.

Herein, we report a pediatric case of anaphylaxis caused by ingestion of edible jellyfish with no previous history of stings or frequent marine sports. The results of the basophil activation test, serum immunoglobulin E (IgE) reactivity, and immunoblot analysis suggested that she was sensitized with jellyfish-specific collagen common to various types of jellyfish by a non-percutaneous route.

## Case presentation

A 14-year-old girl consulted our outpatient department to investigate unknown causative food allergens after two previous episodes of anaphylaxis. When she was 12 years old, 1 hour after oral ingestion of shredded chicken with spicy sesame sauce, which is a type of Chinese food purchased at the supermarket, she experienced abdominal pain, cough, facial skin redness, eyelid edema, and urticaria; thereafter, she was diagnosed with anaphylaxis by the emergency hospital physician and required intramuscular adrenaline administration. Although levels of serum-specific IgE antibodies to suspected causative foods were evaluated, all of which were negative. Serum-specific IgE antibody test for jellyfish were not commercially available in Japan. The skin prick test (SPT) for salted jellyfish was not done at that time because jellyfish consumption was not suspected to be the cause. She was prescribed an adrenaline auto-injector in preparation for accidental anaphylaxis and was followed up at an outpatient clinic. At the age of 13, one hour after oral ingestion of Japanese-style chilled noodles purchased at a convenience store, she developed cough, rhinorrhea, dyspnea, cyanosis, abdominal pain, conjunctivitis, and generalized skin erythema. She was diagnosed with anaphylaxis by the emergency hospital physician, and intramuscular adrenaline administration with oxygen inhalation was required.

She had no significant medical history other than allergic rhinitis or conjunctivitis. She had no history of atopic dermatitis, bronchial asthma, or drug allergies. Although there was a case report about the cross-reactivity between edible jellyfish and fermented soybeans [6], she was able to consume them without any problems. Her family had no history of allergic disease. She used to swim in the ocean once a year; however, she had no experience in marine sports such as surfing or with jellyfish stings. She also had no history of using cosmetics containing jellyfish collagen. She had previously consumed a meal containing edible jellyfish twice when she was approximately 10 years old.

Her diet diaries revealed that edible jellyfish, which she consumed rarely, was common to the meals in the two episodes of anaphylaxis. She had consumed other foods at the time of the reaction without any incident occurring. We contacted each food manufacturer (shredded chicken with spicy sesame sauce and Japanese-style chilled noodles) about the types of jellyfish that contributed to these two episodes, but they could not identify the types. Her blood tests showed a normal blood eosinophil count and elevated serum level of total IgE (498 IU/mL). No positive results were observed for serum-specific IgE antibodies against food-related allergens in either of the two previous episodes of anaphylaxis (egg white, milk, wheat, soybeans, pork, chicken, sesame, and gelatin). To identify the causative food in these two episodes, SPT was performed using five types of commercially available edible jellyfish products (*Rhopilema esculentum, Stomolophus meleagris, Rhopilema hisphidum, Nemopilema nomurai,* and *Lobonema smithi*), fermented soybeans, and their mucilage. The same jellyfish that caused anaphylaxis were not available during the winter season at the time. The reaction to *Rhopilema hisphidum, Nemopilema nomurai,* and *Lobonema smithi* was positive at 15 min with wheal diameters of 5 × 5, 5 × 5, and 10 × 5 mm, respectively, and the reaction to *Rhopilema esculentum*, *Stomolophus meleagris*, fermented soybeans, and mucilage were negative. Histamine hydrochloride (10 mg/mL) and saline were used as positive and negative controls (wheal diameters of 7 × 6 and 0 × 0 mm, respectively).

An oral food challenge test for jellyfish was also conducted with written consent obtained from the patient and her parents. The same Japanese chilled noodles were purchased again in the summer, and edible jellyfish were used for the test. Seventy-five minutes after ingesting a total of 14 g of jellyfish (2 g, 4 g, 8 g every 30 min) dyspnea, cough, wheezing, erythema of the trunk, repetitive vomiting, and drowsiness appeared with hypotension (88/50 mmHg), poor oxygenation (90%), and tachycardia (110/min). Shortly after ingesting the edible jellyfish, the patient had generalized cutaneous erythema, severe respiratory and gastrointestinal symptoms, decreased blood pressure, and tachycardia. She was diagnosed with anaphylaxis according to the diagnostic criteria established by the World Allergy Organization guidelines for the assessment and management of anaphylaxis [14]. She was treated with intramuscular adrenaline injection, volume resuscitation, oxygen inhalation, intravenous infusions of corticosteroids and antihistamines, and bronchodilator inhalation. These treatments resulted in improvement of her symptoms without a biphasic reaction. With the elimination of all kinds of edible jellyfish from her diet, she did not experience any further episodes of anaphylaxis during the subsequent 2-year follow-up period.

**Fig. 1:**
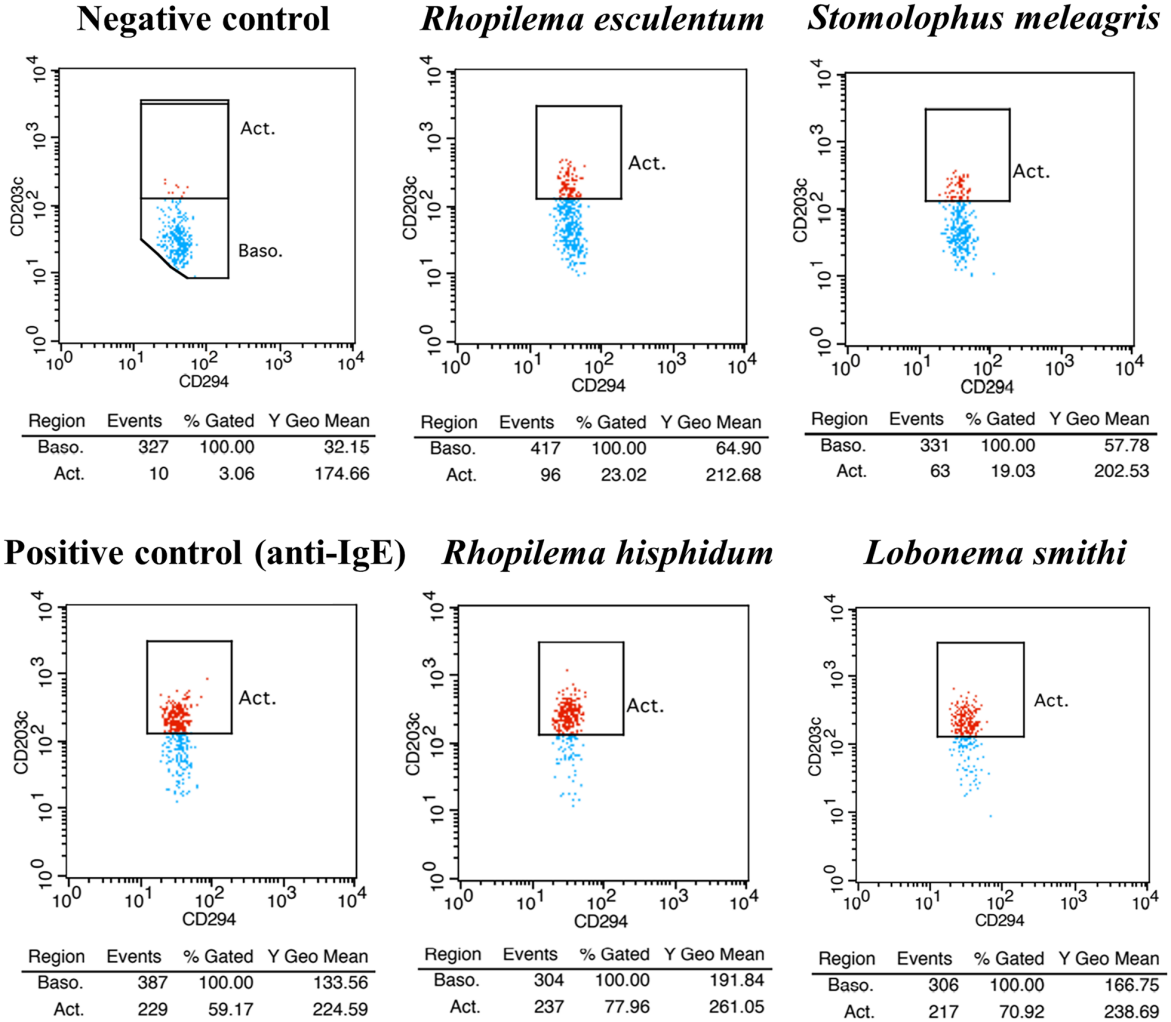
Whole-blood basophil activation tests stimulated with four types of jellyfish extracts. Four types of edible jellyfish (*Rhopilema esculentum, Stomolophus meleagris, Rhopilema hisphidum,* and *Lobonema smithi*) were prepared for the stimulation. The patient’s whole blood was cultured with each edible jellyfish extract. CD203c-expressing basophils were detected using fluorescence-activated cell sorting. The positive control was incubated with anti-immunoglobulin E antibody

To support whether her symptoms were caused by an IgE-mediated allergic reaction, the basophil activation test (BAT) was performed at an external facility (Bio Medical Laboratories, Saitama, Japan) to quantify activated basophil CD203c expression by culturing the whole blood of the patient with jellyfish antigens. We prepared four types of edible jellyfish (*Rhopilema esculentum, Stomolophus meleagris, Rhopilema hisphidum,* and *Lobonema smithi*) for the stimulation. After crushing each jellyfish in distilled water with an ultrasonic homogenizer, the patient’s peripheral whole blood was incubated with each extracted jellyfish sample, and CD203c-expressing basophils were detected using fluorescence-activated cell sorting. BAT revealed 23.0% CD203c-positive cells in *Rhopilema esculentum,* 19.0% in *Stomolophus meleagris*, 59.2% in *Rhopilema hisphidum*, and 78.0% in *Lobonema smithi* at 1000 µg/mL (Fig. 1). In particular, basophil activation was observed even at a low concentration (100 μg/mL) of *Lobonema smithi* (27.9%). BAT showed 70.9% and 3.1% CD203c-positive cells in the positive (anti-immunoglobulin E) and negative controls, respectively.

**Fig. 2:**
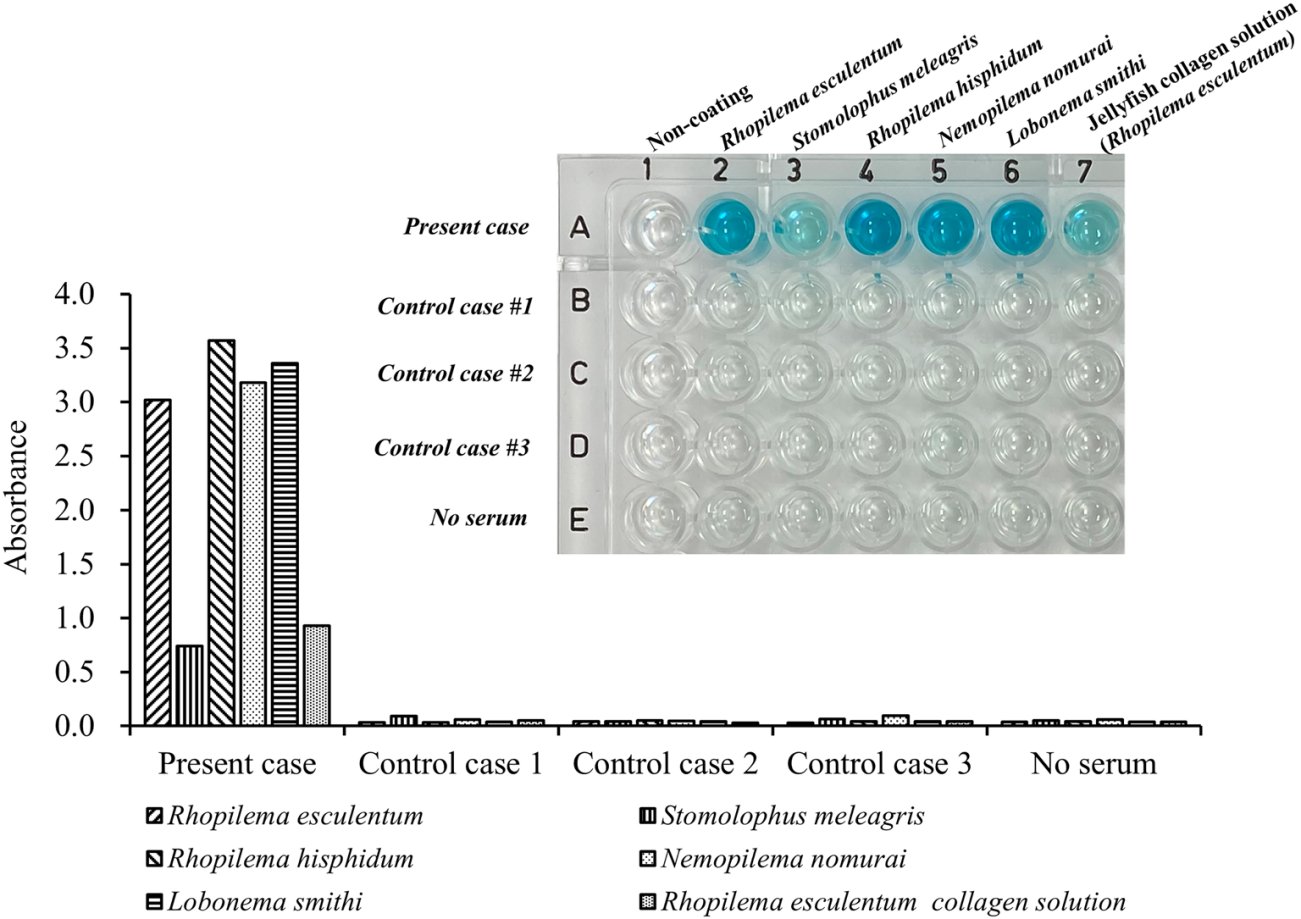
Serum IgE reactivity against purified jellyfish collagen and five types of edible jellyfish extracts. Each well was coated with purified jellyfish collagen solution and five kinds of acid-soluble jellyfish extracts, and serum from the patient or 3 control patients was applied to the appropriate wells for the reaction. The serum jellyfish-specific IgE antibody bound to the coated jellyfish antigen was recognized with an HRP-conjugated mouse anti-human IgE Fc receptor monoclonal antibody. This photo shows the color change to blue in each well due to TMB substrate oxidized by enzymatic reaction. The graphs indicate the absorbance at 450 nm after the color change to yellow after the addition of hydrochloric acid

We also evaluated serum IgE reactivity against jellyfish collagen using enzyme-linked immunosorbent assay (ELISA). We prepared jellyfish extracts, using acid and pepsin, containing collagen from five types of edible jellyfish (*Rhopilema esculentum*, *Stomolophus meleagris*, *Rhopilema hisphidum*, *Nemopilema nomurai,* and *Lobonema smithi*), and a commercially available purified jellyfish collagen solution from *Rhopilema esculentum* (Jellyfish Research Laboratories, Yokohama, Japan), which can be used for coating well plates in cell culture. Each jellyfish was homogenized in 0.5 M acetic acid with 10% pepsin (1: 10,000, derived from porcine gastric mucosa, FUJIFILM Wako Pure Chemical, Osaka, Japan), and the mixture was stirred at 4 °C for 72 h. The supernatant was collected after centrifugation. The protein concentration in each extract was adjusted to 100 µg/mL. An acetic acid and a pepsin solution, which was used for extraction, was used as the background. To coat the bottom of wells with the jellyfish-collagen solution, each solution was added to the appropriate wells of a 96-well plate and incubated at room temperature for 1 h, followed by a wash with phosphate buffered saline (PBS). The patient’s serum was added and incubated overnight at 4 °C. We collected three sera samples from pediatric patients with food allergies other than jellyfish as control sera. Serum of food allergy patients with no history of jellyfish allergy were used as controls. After washing five times with PBS-Tween^®^ 20 (PBS-T), horseradish peroxidase (HRP)-conjugated mouse anti-human IgE Fc receptor monoclonal antibody (1/1000 dilution, Abcam, Cambridge, UK) in 3% bovine serum albumin (BSA) with PBS-T was added, and the mixture was incubated at room temperature for 1 h. After washing five times with PBS-T, 3,3,5,5-tetramethylbenzidine (TMB) substrate was added, the mixture was incubated at 37 °C for 30 min, and the absorbance at 450 nm was measured after the yellow color change after the addition of hydrochloric acid. As a result, serum IgE reactivity against purified jellyfish collagen and extracts from all types of jellyfish was measured (Fig. 2). The levels of IgE reactivity in this patient’s serum were much higher than those in control patients.

**Fig. 3:**
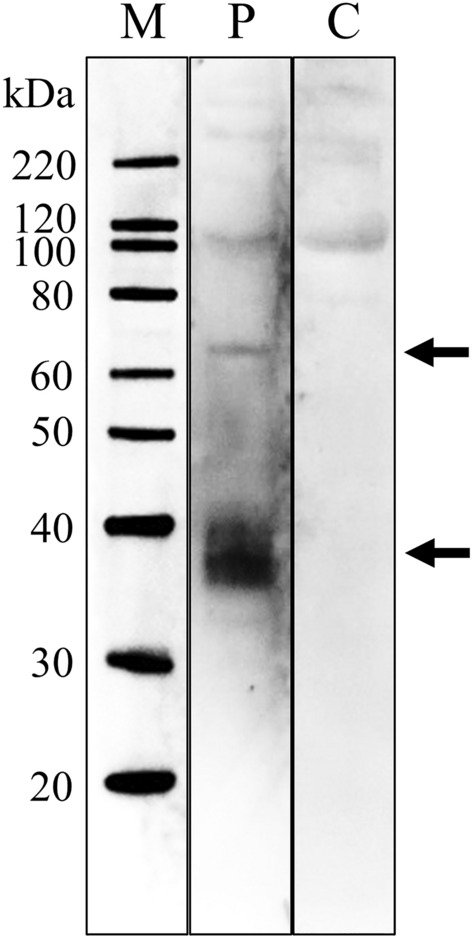
Serum IgE reactivity of patient serum against jellyfish collagen in immunoblot analysis. Purified jellyfish collagen (*Rhopilema esculentum*) was separated using SDS-PAGE, blotted on PVDF membrane, and immuno-probed with the serum from present patient (P) or control allergic patient without jellyfish allergy (C). Lane (M), molecular weight marker

To identify the causative component in the jellyfish collagen, we performed immunoblot analysis for serum IgE reactivity using purified collagen solution from *Rhopilema esculentum* (Jellyfish Research Laboratories, Yokohama, Japan). This jellyfish-collagen solution was more suitable for immunoblot analysis than the salted edible jellyfish because the jellyfish collagen was well solubilized in acid from raw jellyfish. Equal amounts of the acid-soluble jellyfish collagen (15 μg) were separated by sodium dodecyl sulfate–polyacrylamide gel electrophoresis (SDS-PAGE) under denaturing conditions and blotted onto polyvinylidene difluoride (PVDF) membranes. After blocking with 3% BSA in PBS-T, the membrane was incubated overnight with 10% of the patient’s serum. The membranes were then incubated with HRP-conjugated mouse anti-human IgE Fc receptor monoclonal antibody (1/1000 dilution, Abcam), followed by detection with SuperSignal™ West Pico PLUS chemiluminescent substrate system (Thermo Fisher Scientific, Waltham, MA, USA). Immunoblotting analysis showed that the IgE antibodies in the patient’s serum specifically reacted with two protein bands at approximately 40 and 70 kDa of jellyfish collagen unlike those of the control patient’s serum (Fig. 3).

## Discussion and conclusions

We present the case of a 14-year-old girl who had anaphylaxis due to the ingestion of edible jellyfish. Allergic reactions after salted jellyfish ingestion are rare, and including ours, there are seven case reports (Table 1). Of the seven cases, six were Japanese cases, with three pediatric patients under the age of 15. All seven patients developed anaphylaxis after jellyfish ingestion. SPTs using jellyfish for diagnosis were performed in six cases, and all cases were positive.

**Table 1:** Seven cases of allergy due to ingestion of edible jellyfish

Case	Country	Age/sex	Type of jellyfish	Anaphylaxis	Previous jellyfish sting	Skin prick test	Oral food challenge	Basophil activation test	Immuno-blotting	References
1	Japan	32/F	*Rhopilema esculentum*	Yes	Yes (surfer)	*Rhopilema esculentum* (+)	ND	ND	*Aurelia aurita *(200 kDa)	[5]
2	Japan	45/M	Unknown	Yes	Yes (surfer)	Jellyfish (unknown) (+)	ND	Jellyfish (unknown) (+)	ND	[6]
						Poly-γ-glutamic acid (+)				
						Fermented soybeans (+)				
3	Japan	14/M	*Cephea cephea*	Yes	No	*Cephea cephea* (+)	ND	ND	ND	[7]
4	China	26/M	Unknown	Yes	Yes (ND)	ND	ND	ND	ND	[8]
5	Japan	30/M	Unknown	Yes	Yes (fisherman)	*Stomolphus meleagris* (+)	ND	ND	ND	[9]
6	Japan	14/M	*Loboneimoides robustus*	Yes	No	*Loboneimoides robustus* (+)	(+)	ND	ND	[10]
						Poly-γ-glutamic acid (−)				
						Fermented soybeans (−)				
7	Japan	14/F	Unknown	Yes	No	*Rhopilema hisphidum* (+)	(+)	*Rhopilema esculentum* (+)	Collagen from *Rhopilema esculentum *(40 kDa)	Present case
						*Stomolophus meleagris* (−)		*Stomolophus meleagris* (+)		
						*Nemopilema nomurai* (+)		*Rhopilema hisphidum* (+)		
						*Lobonema smithi* (+)		*Lobonema smithi* (+)		
						Fermented soybeans (−)				

*ND* no data

Interestingly, all four adult cases had a previous history of jellyfish stings; two cases were surfers and one was a fisherman, prone to jellyfish stings. Of these cases, an adult case of anaphylaxis due to ingestion of jellyfish also had a delayed allergic reaction to fermented soybeans, and his SPT was positive for fermented soybeans and poly-γ-glutamic acid (PGA), as well as for the causative jellyfish [6]. PGA has been detected in cnidarian nematocyte capsules and is also a major allergen in fermented soybeans [15]. Therefore, transdermal sensitization to PGA due to jellyfish stings in the ocean has been suggested as a cause of allergic reactions due to jellyfish ingestion, which can be cross-reactive to allergy to fermented soybeans [16, 17].

In contrast, two pediatric cases, including our case, developed anaphylaxis due to jellyfish ingestion even with no apparent history of marine sports or jellyfish stabs [7, 10]. None of these patients had a history of fermented soybean allergy, and one of them had a SPTs of PGA and fermented soybeans, both of which were negative. Further, our patient was able to consume fermented soybeans without any problems and had no history of jellyfish stings. In addition, the SPT results for fermented soybeans and their mucilage were also negative, suggesting no PGA sensitization in this case. We speculate that she was sensitized to jellyfish allergens other than PGA, and that her sensitization may have been caused via the gastrointestinal tract by previous ingestion of jellyfish. However, a 7-year-old junior surfer with a history of jellyfish stings reportedly developed fermented soybean-induced late-onset anaphylaxis [17], which suggests that PGA sensitization can be caused by jellyfish sting, even in children.

In Japan, six kinds of edible jellyfish, *Rhopilema esculentum, Stomolophus meleagris, Rhopilema hisphidum, Lobonema smithi*, *Nemopilema nomurai, and Crambionella helmbiru,* are consumed. We confirmed that basophil activation was observed with various edible jellyfish. BAT has emerged as a new diagnostic test for food allergy and has been validated as a quick, reliable, and safe diagnostic tool for the diagnosis of IgE-mediated allergies [18]. BAT can be used to support the results of other diagnostic tests such as serum specific IgE antibody, SPT, and oral food challenge. Our results suggest that in this case, anaphylaxis was caused by an IgE-mediated immediate allergic reaction, regardless of the type of jellyfish. One adult case was reported to be positive in the BAT using a type of jellyfish, but there was no mention of the type of jellyfish [5]. There is no previous case report of BAT performed using various types of jellyfish, as in this case. In this study, the degree of basophil activation differed depending on the type of jellyfish used. It is possible that there are differences in antigenicity depending on the type of jellyfish, but it is also possible that the amount of extracted antigen was different because the salting conditions differed depending on the manufacturer.

Jellyfish is composed of 95% water, and collagen is known to be a major constituent protein of the body of jellyfish, suggesting that collagen may be one of the allergens that causes jellyfish allergy. We were able to measure serum IgE reactivity using ELISA after solubilizing collagen from edible jellyfish with acid and coating assay plate wells with the collagen similar to the collagen coating method used in cell culture. As a result, we confirmed that serum IgE reactivity was observed in purified jellyfish collagen and in various edible jellyfish extracts containing acid-soluble collagen. This result suggests that this patient may be sensitized to jellyfish collagen common to various types of jellyfish. IgE reactivity of *Stomolophus meleagris* was relatively lower than those of the other four types of jellyfish, which may be caused by the difference in the amount of acid-extracted antigen from each salted jellyfish.

A previous case report on PGA sensitization showed that proteins with molecular weights of approximately 200 kDa in jellyfish extracts might be the causative allergens [5]. In this patient, immunoblot analysis revealed proteins at approximately 40 and 70 kDa in the purified jellyfish collagen, which is not consistent with a previous report. Since this case had no suggestion of PGA sensitization, the causative antigen in this case may be different from previously reported cases. We are now considering future studies on the detailed analysis of these proteins.

In conclusion, we report a pediatric patient with anaphylaxis caused by jellyfish ingestion. This patient might have been sensitized to jellyfish-specific collagen, which is common to various types of jellyfish, through a non-percutaneous route. Anaphylaxis of unknown cause might include jellyfish allergy; therefore, clinicians should consider that jellyfish can be a causative food, particularly in Asian people with a dietary culture that consumes edible jellyfish. Although history of jellyfish sting might be an important allergic factor for individuals who consume jellyfish, we should be aware of the possibility of anaphylactic reactions due to jellyfish ingestion even without a history of stings. Anaphylaxis caused by jellyfish ingestion are thought to involve multiple allergens such as PGA or jellyfish-collagen via different sensitization routes; therefore, further detailed investigations are needed in the future.

## Data Availability

Data sharing is not applicable to this article, as no datasets were generated or analyzed during this case report.

## Abbreviations

BAT: Basophil activation test
BSA: Bovine serum albumin
ELISA: Enzyme-linked immunosorbent assay
HRP: Horseradish peroxidase
IgE: Immunoglobulin E
PAGE: Polyacrylamide gel electrophoresis
PBS: Phosphate buffered saline
PGA: Poly-γ-glutamic acid
PVDF: Polyvinylidene difluoride
SDS: Sodium dodecyl sulfate
SPT: Skin-prick test
TMB: Tetramethylbenzidine
